# Specific elastin degradation products are associated with poor outcome in the ECLIPSE COPD cohort

**DOI:** 10.1038/s41598-019-40785-2

**Published:** 2019-03-11

**Authors:** Sarah Rank Rønnow, Lasse Løcke Langholm, Jannie Marie Bülow Sand, Jeppe Thorlacius-Ussing, Diana Julie Leeming, Tina Manon-Jensen, Ruth Tal-Singer, Bruce E. Miller, Morten Asser Karsdal, Jørgen Vestbo

**Affiliations:** 1 Nordic Bioscience A/S, Herlev, Denmark; 2 University of Southern Denmark, The Faculty of Health Science, Odense, Denmark; 3 University of Copenhagen, Copenhagen, Denmark; 4 GSK R&D, Collegeville, PA USA; 5 Division of Infection Immunity and Respiratory Medicine, The University of Manchester, Manchester Academic Health Science Centre, and Manchester University NHS Foundation Trust, Manchester, England

## Abstract

Chronic obstructive pulmonary disease (COPD) is characterized by a slow heterogeneous progression. Therefore, improved biomarkers that can accurately identify patients with the highest likelihood of progression and therefore the ability to benefit from a given treatment, are needed. Elastin is an essential structural protein of the lungs. In this study, we investigated whether elastin degradation products generated by the enzymes proteinase 3, cathepsin G, neutrophil elastase, MMP7 or MMP9/12 were prognostic biomarkers for COPD-related outcomes. The elastin degradome was assessed in a subpopulation (n = 1307) of the Evaluation of COPD Longitudinally to Identify Predictive Surrogate End-points (ECLIPSE) cohort with 3 years of clinical follow-up. Elastin degraded by proteinase 3 could distinguish between COPD participants and non-smoking controls (p = 0.0006). A total of 30 participants (3%) died over the 3 years of observation. After adjusting for confounders, plasma levels of elastin degraded by proteinase 3 and cathepsin G were independently associated with mortality outcome with a hazard ratio per 1 SD of 1.49 (95%CI 1.24–1.80, p < 0.0001) and 1.31 (95%CI 1.10–1.57, p = 0.0029), respectively. Assessing the elastin degradome demonstrated that specific elastin degradation fragments have potential utility as biomarkers identifying subtypes of COPD patients at risk of poor prognosis and supports further exploration in confirmatory studies.

## Introduction

Disease progression of chronic obstructive pulmonary disease (COPD) is slow and very heterogeneous, this is most likely consequent to different phenotypes with different disease trajectories that should be treated individually^1,2^. Phase III studies in COPD are long and costly, and consequently, there is a medical need to develop new and improved biomarkers that accurately identify COPD patients who progress within a short time period, consequent to a given disease phenotype which may be pharmaceutically attenuated. This is essential for the execution of improved phase II clinical studies that will allow confident phase III decision based on actual effects on forced expiratory volume in the first second (FEV_1_)^3,4^.

Elastin is an essential structural protein of the lungs and is responsible for tissue elasticity^5,6^. Loss of the elasticity and elastin content during pathological situations is reported in inflammatory diseases including COPD with co-existing emphysema^7–11^. Tropoelastin, the monomeric form of elastin, has a unique structure that is composed of highly cross-linked and extremely hydrophobic domains, which renders it resistant to proteolytic degradation in healthy conditions^12,13^. Under pathological conditions such as COPD increased numbers of inflammatory cells and fibroblasts leads to an up-regulation of proteases including serine proteinases and matrix metalloproteinases (MMPs)^14^. Both excessive serine proteinase and MMP activity are associated with the destruction of elastin, resulting in specific pathological protein fragments and loss of lung elasticity^11,15^. These proteolytically processed fragments also referred to as neoepitopes are released into the circulation and may be assessed as simple non-invasive biomarkers. These neoepitopes represent a unique fingerprint of proteolytic cleavage of the protein and may be used to identify whether the tissue is pathologically affected^16,17^. Neoepitopes have been proven to be more accurate predictors of disease than their unmodified intact mature protein^18,19^, since measurement of different fragments from the same protein has yielded different information^19–21^. For example, such a fragment is produced when elastin is degraded by neutrophils elastase which may be assessed as a biomarker (EL-NE) associated with chronic inflammation^22^ and emphysema^23^. Such a fragment can also be produced by MMP-7 (ELM7) associated with lung remodeling in IPF^10^, or by MMP9/12 (ELM12) elevated during acute myocardial infarction^24^. In direct alignment, markers of elastin degraded predominantly by the serine proteinases, proteinase 3 (ELP-3) and cathepsin G (EL-CG), are also the result of specific elastin degradation providing relations to other pathological events in lung diseases^25^.

We evaluated aspects of degraded elastin by five different proteinases in a subpopulation in the Evaluation of COPD Longitudinal to Identify Predictive Surrogate End-points (ECLIPSE) cohort. We hypothesized that different elastin fragments would provide complementary pathophysiological information with the hypothesis that MMP, neutrophil elastase, proteinase 3 and cathepsin G activity may play different roles in lung tissue damage in COPD. We also tested the hypothesis that these fragments were prognostic of poor clinical outcomes: a decline in lung function and mortality.

## Results

### Elastin fragments have different pathological specificity

**Figure 1:**
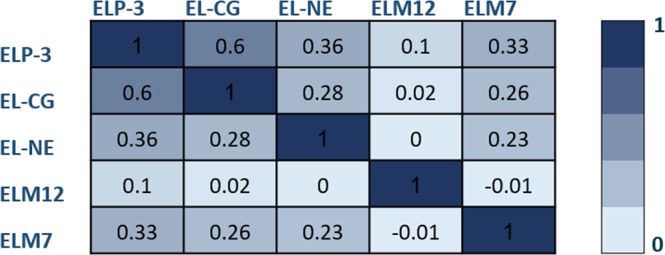
Spearman’s coefficient of rank correlations is listed in the table between the elastin biomarkers. The darkest color represents the most correlated biomarkers.

The five unique elastin neoepitope biomarkers investigated in this study were generated from either cleavage of human elastin by proteinase 3 (ELP-3), cathepsin G (EL-CG), neutrophil elastase (EL-NE), MMP7 (ELM7) or MMP9/12 (ELM12). ELP-3 correlated positively with EL-CG, EL-NE and ELM7 (0.6; 0.36; 0.33), respectively. No significant correlation was observed for any of the other neoepitope biomarkers (Fig. 1).

**Figure 2:**
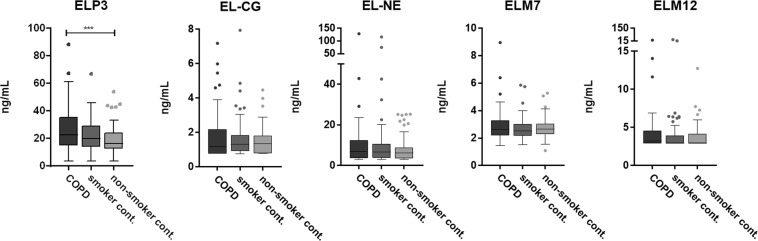
Serological elastin neo-epitope biomarker levels in age, gender and BMI matched COPD (n = 100), smoker controls (n = 99) and non-smoker controls (n = 98). ELP-3 was significantly up-regulated in COPD patient compared to non-smoker controls. Data were analyzed using Kruskal-Wallis test and presented as a Tukey box plot. Asterisks indicate statistical significance *p < 0.05; **p < 0.01; ***p < 0.001; ****p < 0.0001.

Baseline characteristics of smoking controls, non-smoking controls and a subgroup of age, BMI and gender-matched COPD participants are listed in Table 1. As expected, COPD subjects had lower FEV_1_ and FEV_1_/FVC ratio compared to both control groups. Plasma ELP-3 level was significantly up-regulated in COPD when compared to levels of non-smoking controls (p = 0.0004), but not when compared to smoking controls (Fig. 2**)**. No significant difference was observed for EL-CG, EL-NE, ELM7 and ELM12. Furthermore, no correlation between the elastin fragments and FEV_1_ or degree of emphysema defined as more than 10% of lung volume with a density of −950 Hounsfield units on inspiratory computed tomography was observed (data not shown).

**Table 1:** Population demographics and characteristics at baseline.

Characteristic	COPD (n = 100)	Smoker controls (n = 99)	non-smoker controls (n = 98)	P-value
Age (yr)	60 ± 7	60 ± 7	59 ± 7	0.68
Female sex	50 (50)	49 (49)	49 (50)	0.99
Body-mass-index	27 ± 5.7	27 ± 4.3	28 ± 4.5	0.14
Smoking status				
Current smoker	50 (50)	99 (100)	0 (0)	0.002
Smoking history (pack-yr)	40 ± 18	32 ± 17	0 ± 0	<0.0001
Clinical variables				
FEV_1_ (L)	1.46 ± 0.6	3.08 ± 0.8	3.18 ± 0.8	<0.0001
FEV_1_ (% predicted)	48 ± 16	103 ± 15	110 ± 15	<0.0001
FEV_1_/FVC	0.47 ± 0.12	0.75 ± 0.06	0.78 ± 0.05	<0.0001
ELP-3	26.34 ± 15.96	21.39 ± 11.78	18.14 ± 10.30	0.0009
EL-CG	1.76 ± 1.46	2.25 ± 6.60	1.45 ± 0.71	0.83
EL-NE	10.31 ± 14.11	10.22 ± 14.72	7.79 ± 5.64	0.36
ELM7	2.80 ± 1.01	2.65 ± 0.76	2.72 ± 0.67	0.46
ELM12	4.21 ± 2.83	4.01 ± 3.37	3.70 ± 1.46	0.20

Data are shown as mean ± SD, median (25th; 75th) or number (%). FEV_1_, post- bronchodilator forced expiratory volume in 1 second; FVC; forced vital capacity. Statistical significance was determined using Kruskal-Wallis test or chi-squared test.

### Elastin degradation by proteinase 3 and cathepsin G is related to mortality

**Figure 3:**
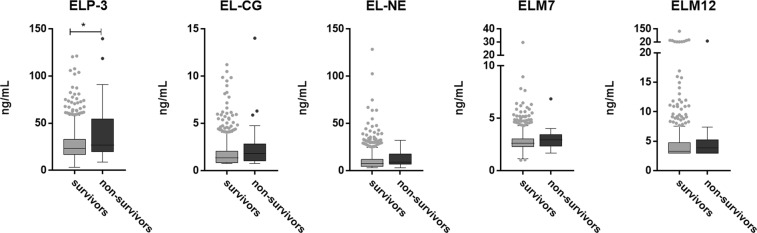
Serological elastin neo-epitope biomarker levels in survivors (n = 970) and non-survivors (n = 30). ELP-3 was significantly elevated in non-survivors compared to survivors. Data were analyzed using Mann-Whitney test and presented as a Tukey box plot. Asterisks indicate statistical significance *p < 0.05; **p < 0.01; ***p < 0.001; ****p < 0.0001.

**Figure 4:**
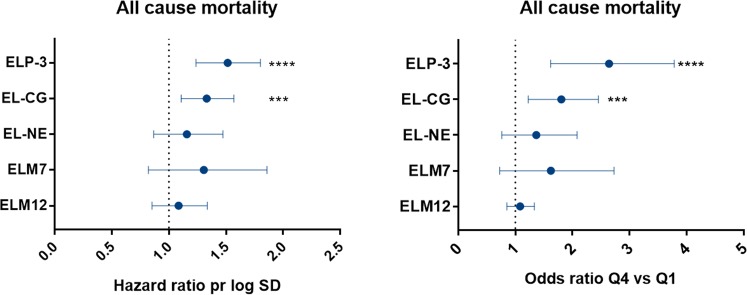
Cox proportional hazard ratio on the left figure and odds ratio for patients belonging to biomarker quartile 4 vs 1 on the right figure. Data are shown as mean (95% CI) hazard ratio for 1 log SD increase in biomarker for all-cause mortality and adjusted for age, smoking status, BODE index, mMRC dyspnea score, inhaled corticosteroids and number of exacerbations the previous year of study start. Asterisks indicate statistical significance *p < 0.05; **p < 0.01; ***p < 0.001; ****p < 0.0001.

Baseline characteristics for survivors and non-survivors are listed in Table 2. Non-survivors were significantly older, had increased mMRC dyspnea score, a higher number of previous exacerbations, a lower use of inhaled corticosteroids, and fewer subjects were current smokers (Table 2). A total of 30 (3%) out of the 1000 COPD participants assessed died over the three years of observation. ELP-3 was significantly increased in non-survivors compared to survivors (p = 0.0202). No significant increase in EL-CG, EL-NE, ELM7 and ELM12 was observed (Fig. 3). After adjusting for relevant covariates, plasma levels of ELP-3 and EL-CG were independently associated with mortality with a hazard ratio per 1 SD increase in biomarker level of 1.49 [95%CI 1.24–1.80, p < 0.0001] and 1.31 [95%CI 1.10–1.57, p = 0.0029], respectively. Moreover, in adjusted analysis, the odds ratio for belonging to the highest quartile as compared to the lowest quartile was significantly associated with all-cause mortality for ELP-3 and EL-CG (2.52 [95%CI 1.62–3.79, p < 0.0001] and 1.74 [95%CI 1.22–2.46, p = 0.0019], respectively) (Fig. 4).

**Table 2:** Population demographics and characteristics at baseline.

Characteristic	Survivors (n = 970)	Non-survivors (n = 30)	P-value
Age (yr)	63 ± 7	68 ± 6	P < 0.0001
Female sex	351 (36)	11 (37)	P = 0.14
Body-mass-index	27 ± 5.8	28 ± 7.4	P = 0.80
Smoking status			
Current smoker	364 (38)	3 (10)	P = 0.002
Smoking history (pack-yr)	47 ± 25	56 ± 42	P = 0.92
Clinical variables			
FEV_1_ (L)	1.42 ± 0.5	1.35 ± 0.5	P = 0.41
FEV_1_ (% predicted)	46 ± 15	46 ± 13	P = 0.99
GOLD stage			P = 0.708
II	480 (50)	14 (47)	
III	390 (40)	14 (47)	
IV	98 (10)	2 (6.7)	
Number of previous exacerbations			P = 0.0170
0	432 (45)	10 (33)	
1	244 (25)	12 (40)	
2	144 (15)	0 (0)	
>2	148 (15)	8 (27)	
mMRC dyspnea score	1 (1;2)	2 (1;3)	P = 0.023
%LLA	16 ± 11	17 ± 11	P = 0.45
BODE index	3 (1;4)	3.5 (2;5)	P = 0.063
Treatments			
Inhaled corticosteroids	139 (14)	3 (10)	P = 0.048
Systemic corticosteroids	8 (0.8)	0	P = 0.77
Statins	237 (24)	6 (20)	P = 0.37
Biomarkers			
ELP-3	26.8 ± 15.7	41.7 ± 32.7	P = 0.020
EL-CG	1.7 ± 1.3	2.6 ± 2.6	P = 0.066
EL-NE	10.1 ± 9.7	11.8 ± 8.8	P = 0.16
ELM7	2.8 ± 0.9	3.0 ± 1.0	P = 0.16
ELM12	4.5 ± 5.5	5.1 ± 5.4	P = 0.22

Data are shown as mean ± SD, median (25th; 75th) or number (%). FEV_1_, post-bronchodilator forced expiratory volume in 1 second; GOLD, global initiative for chronic obstructive lung disease; mMRC, modified Medical Research Council dyspnea scale; %LLA, percent low areas of attenuation below 950 Hounsfield unit; BODE, BMI, airflow obstruction, dyspnea and exercise capacity index. Statistical significance was determined using Mann-Whitney U test or chi-squared test.

## Discussion

There is a medical need for developing new and improved biomarkers to identify COPD subjects with a rapid disease progression, who potentially have a higher chance of benefitting from a symptom modifying COPD treatment. Here we investigated elastin degradation fragments, so-called neoepitopes, as blood-based biomarkers of disease progression for COPD in a non-invasive manner. We found that elastin degraded by protease 3 and cathepsin G, was associated with a higher risk of mortality in subjects with COPD.

Elastin is a unique signature protein of the lungs. Consequently, biomarkers generated from elastin could be associated with higher tissue accuracy and pathophysiological relevance; although these neoepitopes could potentially arrive from other organs than the lung. The importance of proteolytic elastin degradation in COPD is highlighted by the observation that exacerbations in COPD are associated with accelerated elastin turnover^4,10^. Desmosine and isodesmosine have long been proposed as biomarkers of lung tissue destruction as they are the crosslinking elements of elastin, released during lung tissue destruction^26^. It has been demonstrated that subjects with COPD have higher levels of plasma desmosine and that levels are able to predict mortality^27^ which is in concordance with our findings. Moreover, a study with 349 subjects with one-third suffering from COPD showed that urinary desmosine was significantly correlated with all lung function measures^28^. This underlines the importance of elastin degradation as a potential biomarker in COPD, however, the desmosine technology identifies non-specific elastin fragments that are being generated through many different processes, both physiological and pathophysiological. The technique and biomarkers used in the present study allowed for investigation of elastin degradation predominantly produced by specific proteinases, known to be up-regulated in respiratory diseases and therefore more carefully quantify the aspects of the elastin degradome^10,22–24^.

Plasma levels of ELP-3 and EL-CG were associated with all-cause mortality in COPD implying that proteinase 3 and cathepsin G play a significant role in COPD. During the progression of COPD, inflammatory cells infiltrate the lungs, which has been shown to release proteinase 3, cathepsin G and neutrophil elastase into the lungs^29^. These proteinases are known to efficiently degrade elastin^30^, which is in concordance with the results from this study where a higher degree of ELP-3 or EL-CG was significantly associated with a poor outcome. Moreover, proteinase 3 activity was present in the sputum of COPD subjects in a higher amount than the activity of neutrophil elastase implicating a bigger role for proteinase 3 in COPD than previously thought^31^. This may in part explain why ELP-3 demonstrated a better association with outcomes relative to EL-NE. Since plasma levels of ELP-3 and EL-CG were associated with all-cause mortality, a correlation with FEV_1_ might have been expected, as this was previously observed for desmosine^28^. In general, the FEV_1_ decline for the subpopulation of ECLIPSE studied here were associated with a considerable intra and inter-person variability which makes an evaluation of predictive biomarkers for lung function decline challenging, 31% improved or slightly decreased in FEV_1_ while only 38% participants in the entire cohort demonstrated significant FEV_1_ decline^32^.

The importance of unique elastin fragments has been emphasized by the fact that they can act as chemotactic peptide for different cell types, showing their ability to function as matrikines^33^. The hexapeptide VGVAPG within tropoelastin is well known for its chemotactic activity attracting monocytes and fibroblasts and its ability to regulate MMP expression and activity^34,35^. Likewise, other fragments of elastin corresponding to XGXXPG, where X is a generic hydrophobic residue, has also been shown to be active peptides^35–37^. In fact, the ELM12 fragment holds the sequence VGVAPG in its peptide, which indicates that it might be a matrikine, however, in the current study, no pathological relevance of this biomarker was observed.

The emphysema phenotype, such as the multi-organ loss of tissue (MOLT), is currently receiving increased attention, and high elastin turnover could be associated with it, which has been shown by others^23,38,39^. In accordance with our findings, another study measuring plasma desmosine in the ECLIPSE cohort was not able to show a relationship between emphysema and elastin degradation^27^. The lack of association could be explained by the fact that COPD participants from the ECLIPSE cohort had an established disease (GOLD II-IV) with a stable state during sampling. This could result in less elastin present in their lungs or a lower disease activity than during an exacerbation, which could explain why elastin degradation is not associated or increased in subjects with emphysema in this cohort.

The limitations of this study include the low number of deceased subjects, even though we were able to detect a significant association with all-cause mortality for the two biomarkers ELP-3 and EL-CG. To generalize these finding to a more general COPD population they have to be confirmed in secondary cohorts. Furthermore, using the subpopulation of the full ECLIPSE study comprising the study participant that progress the least and most in terms of FEV_1_ decline during the study period might explain the low number and make a less optimal subpopulation to study mortality. In addition, the ELP-3, EL-CG, ELM7 and ELM12 was measured in heparin plasma at year 1, whereas EL-NE was measured in 6-month serum. This could create some difficulties in directly comparing the results.

## Conclusion

In conclusion, we have demonstrated that five cleavage-specific fragments of elastin generated by five different proteinases reflect different pathological processes in COPD. ELP-3 and EL-CG demonstrated promise as prognostic biomarkers for all-cause mortality reflecting an increase of elastin remodeling by proteinase 3 and cathepsin G. This study demonstrated the importance of evaluating elastin turnover in the pathology and natural history of COPD.

## Methods

### Study design and participants

The analysis was based on the three-year observational longitudinal study ECLIPSE (ClinicalTrials.gov. number, NCT00292552), described previously^32,40^. The full ECLIPSE study included 2163 participants with COPD. The enrollment criteria included an FEV_1_ of less than 80% of the predicted value and a FEV_1_/forced vital capacity (FVC) ratio of 0.7 or less assessed after the use of bronchodilators. COPD participants had a smoking history of 10 or more pack-years. 343 smoking controls with a smoking history of 10 or more pack-years and 223 nonsmoking controls were also included in the study. The controls had an FEV_1_ of more than 85% of the predicted value and an FEV_1_/FVC ratio of 0.7 or more after the use of bronchodilators. Control participants had to be free of significant comorbidities, determined from screening investigation, physical examination and medical history. Eight study visits were conducted at baseline, month three, six and subsequently every six months over three years. The current analysis was performed on 1307 participants consisting of 1000 COPD, 207 smoking controls and 100 non-smoking controls. All-cause mortality was recorded until year 3. The study was conducted according to the Declaration of Helsinki and Good Clinical Practice guidelines and was approved by relevant ethics and review boards (Supplementary Table 1). Participants provided informed consent.

### Quantification of serological biochemical biomarkers

**Figure 5:**
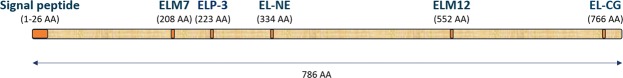
Schematic figure of tropoelastin and the location of the five unique elastin neoepitope biomarkers generated from either cleavage of human elastin by proteinase 3 (ELP-3), cathepsin G (EL-CG), neutrophil elastase (EL-NE), MMP7 (ELM7) or MMP9/12 (ELM12).

Whole blood was collected from fasting participants and transferred to vacutainers containing sodium heparin. Plasma was obtained by centrifugation of vacutainer tubes at 2,000 g for 10–15 minutes and stored at −80 °C until analysis. Elastin degradation by proteinase 3 (ELP-3), cathepsin G (EL-CG) and MMP9/12 (ELM12) was measured in heparin plasma obtained from 1307 study participants at the year 1 visit using well-validated ELISAs each utilizing monoclonal antibodies targeting a specific neoepitope (Nordic Bioscience, Herlev, Denmark), see specifications in Table 3. Measurements were performed in a blinded manner according to the instruction of the manufactures. Previously, elastin degradation by neutrophil elastase (EL-NE) was measured in month six serum samples whereas elastin degraded by MMP7 (ELM7) was measured in year 1 heparin plasma^41^. The five unique elastin neoepitope biomarkers investigated in this study ELP-3, EL-CG, EL-NE, ELM7 and ELM12 originates from locations throughout the tropoelastin **(**Fig. 5**)** and are very different in terms of activation, inhibition, substrate specificity and source (Table 4**)**.

**Table 3:** Biomarker specifications.

Biomarker	Specification	Sequence	Location	Reference
ELP-3	Elastin degraded by proteinase 3	LPGGYGLPYT	213–222	^25^
EL-CG	Elastin degraded by cathepsin G	LGGVAARPGF	756–765	^25^
EL-NE	Elastin degraded by neutrophil elastase	GGPGFGPGVV	325–334	^22^
ELM7	Elastin degraded by MMP7	IKAPKLPGGY	208–217	^15^
ELM12	Elastin degraded by MMP12 or MMP9	GVAPGIGPGG	543–552	^42^

**Table 4:** Proteinase characteristics.

Proteinase	Substrate specificity	Optimal pH for activity	Source	Activation	Activation enzyme	Inhibitors
Proteinase 3	Small hydrophobic residues at P1: Val, Cys, Ala, Met, Leu, Ser	~8.0	Neutrophils Monocytes Basophils	Cleavage of N-terminal signal peptide and then cleavage of N-terminal prodipeptide = > enzymatic activity and the C-terminal	Dipeptidyl peptidase I	α2-macroglobulins, α1-PI, SerpinB1, α1-ACT, Elafin, Eglin c
Cathepsin G	Aromatic or positively charged residue at P1: Phe, Tyr, Lys, Arg	~7.5	Neutrophils Monocytes Mastocytes	Cleavage of N-terminal signal peptide and then cleavage of N-terminal prodipeptide = > enzymatic activity and the C-terminal	Dipeptidyl peptidase I	β-ketophosphonic acids, aminoalkylphosphonic esters and boswellic acids
Neutrophil elastase	Small hydrophobic residues at P1: Val, Cys, Ala, Met, Ile, Leu, Ser	8.0–8.5	Neutrophils Monocytes	Cleavage of N-terminal signal peptide and then cleavage of N-terminal prodipeptide = > enzymatic activity and the C-terminal	Dipeptidyl peptidase I	α2-macroglobulins, α1-PI, SerpinB1, α1-ACT, Elafin, Eglin c
MMP7	Acidic residues P1: Leu, Ile, Val, Met	7.0	Macrophages lymphocytes	Proteolytic cleavage resulting in removal of the prodomain, final step is autolytic cleavage at Glu77↓Tyr78	Trypsin, plasmin, MMp-3, MMP10 and others	TIMPs, 1,10-phenanthroline, DTT
MMP9	Small residues P1: Gly, Ala, Ser	7.5	Macrophages lymphocytes	Proteolytic removal of the N-terminal propeptide involving a cysteine switch mechanism	Matrilysin, interstitial collagenase, tissue kallikrein, plasmin, macrophage elastase	TIMPs
MMP12	P1: Leu,	8.0	Macrophages	Protelytic removal of the prodomain and the C-terminal	Trypsin, plasmin, neutrophil elastase, stromelysin-1	TIMP-1, α_2_-macroglobulin, 1,10-phenanthroline

### Statistical analysis

Population demographics were compared using Kruskal-Wallis test, Mann-Whitney U test and chi-squared test as appropriate. Kruskal-Wallis test and Mann-Whitney test were used to compare biomarker levels between COPD subjects, controls, survivors and non-survivors. Cox proportional hazard regression was used to assess the prognostic value of each biomarker for all-cause mortality for one standard deviation (SD) increase in biomarker level. Logistic regression was used to find the odds ratio for all-cause mortality belonging to the upper quartile versus the lower quartile. The risk of death was assessed adjusted for confounders that were significantly different between survivors and non-survivors. The covariates adjusted for were age, smoking status, mMRC dyspnea score, use of inhaled corticosteroids and number of exacerbations in the year prior to blood sampling.

The software MedCalc (MedCalc version 14.8.1, MedCalc software bvba, Ostend, Belgium) was used to perform all statistical analysis.

## Supplementary information

Ethics and review boards

## Data Availability

The dataset generated during and analyzed during the current study are available from the corresponding author on reasonable request.
