# Supplementary material for: Specific elastin degradation products are associated with poor outcome in the ECLIPSE COPD cohort
Source: Sci Rep. 2019 Mar 11;9:4064. doi: 10.1038/s41598-019-40785-2 (PMC6412140; doi:10.1038/s41598-019-40785-2)
Supplement: Supplementary file 1 — Ethics and review boards [file 41598_2019_40785_MOESM1_ESM.pdf]

Elastin degradation is not just elastin degradation: Results from 5 different biomarkers of elastin in the ECLIPSE cohort

Authors: Sarah Rank Rønnow[1,2*], MSc; Lasse Løcke Langholm[1,3], MSc; Jannie Marie Bülow Sand[1], PhD; Jeppe Thorlacius-Ussing[1], BSc; Diana Julie Leeming[1], PhD; Tina Manon-Jensen[1], PhD; Ruth Tal-Singer[4ʹ], PhD; Bruce E. Miller[4], PhD; Morten Asser Karsdal[1], PhD; and Jørgen Vestbo[5ʹ], MD

'The Evaluation of COPD Longitudinally to Identify Surrogate Endpoints (ECLIPSE) study, investigators.
[1]Nordic Bioscience A/S, Herlev, Denmark, [2]University of Southern Denmark, The Faculty of Health Science, Odense, Denmark, [3]University of Copenhagen, [4]GSK R&D, Collegeville, PA, USA, [5]Division of Infection Immunity and Respiratory Medicine, The University of Manchester, Manchester Academic Health Science Centre, and Manchester University NHS Foundation Trust, Manchester, England.

*Correspondence to sar@nordicbio.com

Supplementary

Table 1. Ethics and review boards

Inv/Site No.	Institution & Address	IEC/IRB Committee
027904/023622	Asthma Centre<br>Ivan Vazov Street Nr 31<br>PO Box 1018<br>Pleven, 5800 Bulgaria	Ethics Committee for Multicentre Trials<br>8, Damyan Gruev str.,<br>Sofia 1303<br>Bulgaria
076209/023623	Military Medical Academy<br>Georgi Sofiiski 3 str<br>Sofia 1606 Bulgaria	Ethics Committee for Multicentre Trials<br>8, Damyan Gruev str.,<br>Sofia 1303<br>Bulgaria
006269/023794	Montreal Chest Institute<br>3650 St-Urbain, Room K307<br>Montreal, QC H2X 2P4 Canada	McGill University Health Center<br>Research Ethics Board<br>3650 St Urbain<br>Montreal, QC H2X 2P4
006610/023668	The Lung Center<br>2775 Laurel St., 7th Floor<br>Vancouver, BC V5Z 1M9 Canada	The University of British Columbia<br>Office of Research Services Clinical Research Ethics Board<br>Room210, 828 West 10th Ave<br>Vancouver, BC V5Z 1L8 Canada
004970/023483	Queen Elizabeth II Health Sciences Centre<br>Halifax Infirmary<br>1796 Summer St., Room 5452<br>Halifax, NS B3H 3A7 Canada	Capital Health Research Ethics Board<br>Centre for Clinical Research Building<br>118-5790 University Avenue<br>Halifax, NS B3H 1V7 Canada
029347/023796	McMaster University, Health Sciences Center<br>1200 Main St. West, Room 3U25<br>Hamilton, ON L8N 3Z5 Canada	Hamilton Health Sciences/Faculty of Health Sciences Research Ethics Board<br>293 Wellington St N,<br>Suite 102<br>Hamilton, Ontario L8L 8E7<br>Canada
035031/023795	Pacific Lung Health Center<br>1081 Burrard Street<br>8B Providence Wing<br>Vancouver, BC V6Z 1Y6 Canada	Office of Research Services (ORS)<br>Providence Health Care Research Institute<br>Room 1125, 11th Floor<br>1190 Hornby Street c/o 1081 Burrard Street<br>Vancouver, BC V6Z 1Y6
006193/023547	Hopital Laval, Recherche Clinic<br>Centre de Pneumologie<br>2725 Chemin Sainte Foy<br>Pavillion U, Locale U 1751<br>Sainte Foy, QC G1V 4G5 Canada	Comité d'éthique de la recherche<br>Institut Universitaire de Cardiologie et de Pneumologie de Québec (IUCPQ)<br>2725, chemin Ste-Foy<br>Quebec, Qc<br>Canada<br>G1V 4G5
004395/02409 4	Kingston General Hospital<br>Richardson House<br>102 Stuart Street<br>Kingtson, ON K7L 2V6 Canada	Queens University<br>Office of Research Services<br>Fleming Hall, Jemmett Wing, Room 301<br>Queens University<br>Kingston, ON, Canada
000309/024204	SPLiN s.r.o.<br>Oddeleni TRN<br>Cimicka 37/446<br>Praha 8 18200 Czech Republic	Multicentric Ethics Committee Fakultni nemocnice v Motole<br>V Uvalu 84 Prague 5 ZIP: 150 06<br>Czech Republic
000683/023960	H:S Hvidovre Hospital<br>Hjerte-Lungemedicinsk afdeling<br>Kettegaard Alle 30<br>Opgang 1<br>Hvidovre 2650 Denmark	Den videnskabsetiske komité for region hovedstaden<br>Regionsgaarden<br>Kongensvænge 2<br>3400 Hillerød
001687/024403	Astmacentrum Hornerheide<br>Hornerheide 1<br>Horn 6085 NM Netherlands	METC Zuidwest-Holland<br>M.H.H.A. Kirkels-Breukers<br>P.O Box 5011<br>2600 GA Delft<br>The Netherlands

082272/023579	Haukeland Universitets sykehus<br>Chest department<br>Jonas Liesvei 65<br>Bergen N 5021 Norway	Regional Ethic Committee West<br>Haukeland University Hospital, N-5021 Bergen, Norway
014566/024144	P3 Research Bown Hospital<br>Churchill Drive Crofton Downs<br>Wellington 6035 New Zealand	c/- Ministry of Health<br>1-3 The Terrace<br>Level 1<br>Wellington<br>6011
136098/024146	KOPA Golnik<br>Golnik 36<br>4204 Golnik Slovenia	The National Medical Ethics Committee of the Republic of Slovenia<br>University Institute of Clinical Neurophysiology, Medical Center Ljubljana,<br>Zaloška c. 7,<br>SI-1525 Ljubljana
108244/026658	Hospital Son Dureta<br>C/ Andrea Doria 55<br>Palma de Mallorca 07014 Spain	Comité ètic d´investigació clínica Illes Balears<br>Conselleria de Salut i Consum<br>Direcció General d'Avaluació i Acreditació<br>Comitè Ètic d'Investigació Clínica de les Illes Balears (CEIC-IB)<br>Camí de Jesús, 38 A<br>07011 Palma - Illes Balears
000473/023973	Aintree University Hospitals NHS Foundation Trust<br>Respiratory Research Department<br>Longmoor Lane, Ward 14a<br>Liverpool L9 7AL United Kingdom	Oxfordshire REC C<br>2nd Floor, Astral House<br>Chaucer Business Park<br>Granville Way<br>Bicester OX26 4JT
082424/023706	Cambridge Institute for Medical Research<br>Department of Medicine<br>Hills Road, Wellcome Trust / MRC Building<br>Cambridge CB2 2XY United Kingdom	Oxfordshire REC C<br>2nd Floor, Astral House<br>Chaucer Business Park<br>Granville Way<br>Bicester OX26 4JT
029855/023707	New Royal Infirmary of Edinburgh<br>Little France Crescent, Old Dalkeith Road<br>51 Little France Crescent<br>Edinburgh Midlothian EH16 4SA<br>United Kingdom	Oxfordshire REC C<br>2nd Floor, Astral House<br>Chaucer Business Park<br>Granville Way<br>Bicester OX26 4JT
023731/023974	Wythenshawe Hospital<br>Medicine Evaluation Unit<br>Southmoor Road<br>The Langley Building, North West Lung Research Centre<br>Manchester M23 9LT United Kingdom	Oxfordshire REC C<br>2nd Floor, Astral House<br>Chaucer Business Park<br>Granville Way<br>Bicester OX26 4JT
029742/024037	The Royal Free Hospital<br>Academic Unit of Respiratory Medicine<br>Pond Street<br>London NW3 2QG United Kingdom	Oxfordshire REC C<br>2nd Floor, Astral House<br>Chaucer Business Park<br>Granville Way<br>Bicester OX26 4JT
001069/024393	Institute of Phthisiatry and Pulmonology<br>Department of Pulmonology<br>10, Amosova Str<br>Kiev 03680 Ukraine	
001047/024392	Institute of Phthisiatry and Pulmonology<br>Department of Pulmonology<br>10, Amosova Str<br>Kiev 03680 Ukraine	
001071/024362	Institute of Phthisiatry and Pulmonology<br>Department of Pulmonology<br>10, Amosova Str<br>Kiev 03680 Ukraine	
001063/024364	Donetsk State Medical University<br>Department of Therapy<br>16 Illicha prospect<br>Donetsk 83003 Ukraine	

044783/023140	University of Texas Health Science Center<br>Pulmonary Diseases<br>7400 Merton Minter Blvd., (111E)<br>San Antonio, TX 78229 United States	University of Texas Health Science Center<br>7703 Floyd Curl Drive, Mail Code 7830<br>San Antonio, TX 78229-3900
077534/023146	Rhode Island Hospital<br>Division of Pulmonary, Sleep & Critical Care Medicine<br>593 Eddy Street, APC 7th Floor<br>Providence, RI 02903 United States	Lifespan Office of Research Administration<br>167 Point Street<br>Providence, RI 02903
013075/023147	Los Angeles Biomedical Research Institute at Harbor-UCLA Medical Center<br>Rehab Clinical Trials Center<br>1124 W. Carson St., Bldg. J4<br>Torrance, CA 90502 United States	John F. Wolf, MD Human Subjects Committee<br>Los Angeles Biomedical Research Institute at Harbor-UCLA Medical Center<br>1124 West Carson Street<br>Torrance, CA 90502
008578/023354.	St. Elizabeth's Medical Center<br>Pulmonary STN-3<br>736 Cambridge Street<br>Boston, MA 02135 Unites States	Research/Human Subjects Committee<br>Caritas St. Elizabeth's Medical Center<br>Cambridge St., HOQ3<br>Boston, MA 02135
021992/023148	Pulmonary Associates of Richmond, Inc.<br>1000 Boulders Parkway, Suite 201<br>Richmond, VA 23225 United States	Goodwyn Institution Review Board<br>9380 Main Street<br>Cincinnati, OH 45242
010875/023149	Pulmonary Associates, PA<br>1112 East McDowell Road<br>Phoenix, AZ 85006 United States	Goodwyn Institution Review Board<br>9380 Main Street<br>Cincinnati, OH 45242
011553/023150	Advances in Medicine<br>42362 Bob Hope Drive<br>Rancho Mirage, CA 92270 Unites States	Western International Review Board<br>3535 Seventh Ave SW<br>Olympia, WA 98508
010094/023355	Baylor Clinic-Baylor College of Medicine<br>6620 Main Street<br>Suite 11B, 16<br>Houston, TX 77030 United States	Baylor College of Medicine IRB<br>Clinical Research Studies<br>One Baylor Plaza, Mail stop 600D<br>Houston, TX 77030
015497/023356	Dartmouth-Hitchcock Medical Center<br>Pulmonary & Critical Care Center<br>One Medical Center Drive<br>Lebanon, NH 03756 Unites States	Dartmouth-Hitchcock Medical Center<br>Committee for the Protection of Human Subjects<br>11 Rope Ferry Road #6210<br>Hanover, NH 03755
008005/023357	National Jewish Medical & Research Center<br>Weinberg Clinical Research Unit<br>1400 Jackson Street<br>Denver, CO 80206 United States	National Jewish Medical & Research Center IRB<br>1400 Jackson Street<br>Denver, CO 80206
009021/023358	University of Nebraska Medical Center<br>Pulmonary Clinical Studies Unit<br>982465 Nebraska Medical Center<br>DRC II1022<br>Omaha, NE 68198 United States	University of Nebraska Medical Center IRB<br>Academic & Research Services Bldg. 3000<br>987830 Nebraska Medical Center<br>Omaha, NE 68198
083482/023571	Yale University School of Medicine<br>Internal Medicine/Pulmonary<br>1 Gilbert Street, TAC S 441<br>New Haven, CT 06520 United States	Yale University School of Medicine<br>Human Investigation Committee<br>47 College Street, Suite 204<br>New Haven, CT 06520
015449/023359	Mayo Clinic<br>Pulmonary Clinical Research Center<br>Lanmark 2-46<br>14 – 2nd Street SW<br>Rochester, MN 55905 United States	Mayo Foundation IRB<br>201 Building, Room 4-60<br>200 First Street SW<br>Rochester, MN 55905
080801/023389	Creighton University Medical Center<br>Pulmonary & Critical Care Division<br>601 N. 30th Street, Suite 3820<br>Omaha, NE 68131 United States	Creighton University Medical Center IRB<br>2500 California Plaza<br>Omaha, NE 68178

010532/023489	University of Pittsburgh Medical Center<br>Emphysema Research Center<br>3471 5th Ave., Suite 1211<br>Pittsburgh, PA 15213 United States	University of Pittsburgh IRB<br>3500 Fifth Ave, Ground Level<br>Pittsburgh, PA 15213

021093/023390	Houston VA Medical Center<br>2002 Holcombe Blvd.<br>Pulmonary 111-1, Room 3C-220<br>Houston, TX 77030 United States	Baylor College of Medicine IRB<br>Clinical Research Studies<br>One Baylor Plaza, Mail stop 600D<br>Houston, TX 77030
008864/023392	Midwest Chest Consultants, PC<br>330 First Capital Drive, Suite 470<br>St. Charles, MO 63301 United States	Goodwyn Institution Review Board<br>9380 Main Street<br>Cincinnati, OH 45242
077533/023391	Harvard University-Brigham & Women's Hospital<br>Channing Laboratory<br>181 Longwood Ave.<br>Boston, MA 02115 United States	Brigham & Women's Hospital IRB<br>Partners Human Research Office<br>116 Huntington Ave, Suite 1002<br>Boston, MA 02116
016586/023393	University of Miami School of Medicine<br>1600 NW 10th Ave, #7064-A (R-47)<br>Miami, FL 33136	Western International Review Board<br>3535 Seventh Ave SW<br>Olympia, WA 98508
057745/023394	Johns Hopkins Asthma & Allergy Center<br>5501 Hopkins Bayview Circle, Room 3B-58<br>Baltimore, MD 21224 United States	John Hopkins School of Medicine<br>Office of Human Subject Research<br>1620 McElderry St., Reed Hall<br>Suite B 130<br>Baltimore, MD 21205
012252/023395	St. Francis Hospital & Medical Center<br>Pulmonary Medicine<br>114 Woodland Street<br>Hartford, CT 06105 United States	St. Francis Hospital & Medical Center IRB<br>Department of Research<br>114 Woodland St.<br>Hartford, CT 06105
